# Capsaicin intake and oral carcinogenesis: A systematic review

**DOI:** 10.4317/medoral.24570

**Published:** 2021-02-20

**Authors:** Andrea Mosqueda-Solís, Irene Lafuente-Ibáñez de Mendoza, José Manuel Aguirre-Urizar, Adalberto Mosqueda-Taylor

**Affiliations:** 1 Department of Cell and Molecular Biology, Karolinska Institutet, Stockholm, Sweden; 2 Stomatology II Department, University of the Basque Country (EHU), Leioa, Spain; 3 Health Care Department, Autonomous Metropolitan University Xochimilco, Mexico City, Mexico

## Abstract

**Background:**

Chili is the most heavily and frequently consumed spice, either as a flavouring or colouring agent, and it is also a major source of pro-vitamin A, vitamin E and C. The main capsinoidcapsaicinoid found in chili peppers is capsaicin. It has been demonstrated that capsaicin acts as a cancer-suppressing agent through its antioxidant and anti-inflammatory effects, by blocking several signal transduction pathways. Oral squamous cell carcinoma is one of the most prevalent cancer worldwide. It is noteworthy that in countries where populations of diverse ethnic groups co-exist, differences have been observed in terms of incidence of oral cancer. The variances in their diet could explain, at least in part, these differences. The objective of this systematic review is to explore if there is evidence of a possible relationship between capsaicin intake and the incidence of oral squamous cell carcinoma, and discuss such association.

**Material and Methods:**

A bibliographical search was made in PubMed, Scopus and Web of Science databases, and finally 7 experimental studies were included; OHAT risk of bias tool was used to assess their quality.

**Results:**

allAll the studies confirm that capsaicin is a chemopreventive agent that prevents the development of oral cancer, through inhibition of malignant cell proliferation and increase of apoptosis.

**Conclusions:**

More human studies are needed in order to clarify the real link between consumption of chili (capsaicin) and the prevalence of oral cancer.

** Key words:**Chili, capsaicin, oral epithelial dysplasia, oral cancer, cell proliferation, apoptosis.

## Introduction

Peppers, chilis or Capsicum are versatile crops included in most daily diets, especially in some geographical areas like China, Mexico, Turkey and Indonesia (1). Capsicum plants are tropic crops that grow better in hotter zones and chili peppers are the pungent fruits of various species of the Capsicum genus and members of the Solanaceae family (2).

Chili is the most heavily and frequently consumed spice, as a flavouring or colouring agent, and it is also a major source of pro-vitamin A (carotene), vitamin E (α-tocopherol) and vitamin C (ascorbic acid) (3). Mature pepper fruits are rich in carotenoids with antioxidant properties and have high contents of phenolics, especially flavonoids; in addition, there are reports that mention that they have antioxidant and other bioactive properties, as well as many essential nutrients such as capsinoids (4-5). Capsinoids comprise a distinctive group of molecules in fruits and plants, which display potentially valuable pharmacological and bioactive properties (6). Capsaicin (trans-8-methyl-N-vanillyl-6-nonenamide) is the main capsinoid found in chili peppers (7).

In addition to its use as a major spice and food additive, capsinoids have also been used for medical and therapeutic reasons (8-9). It has been demonstrated that capsaicin also acts as a cancer-suppressing agent through its antioxidant and anti-inflammatory effects, by blocking several signal transduction pathways, including Nf-kB and AP-1 (10-11).

Cancer is a major global public health problem; according to World Health Organization (WHO) it is the second leading cause of death globally (12). The association between poor nutrition and cancer is increasingly evident, and following a healthy diet is a key lifestyle change to reduce its role as a cancer risk factor. Therefore, interventions to reduce smoking, improve diet and increase physical activity must become much higher priorities in the general population’s health and health care systems (13). Furthermore, the intake of specific foods like tomatoes, citric fruits, olive oil, berries, honey, tea, aloe vera or curcuma contain active components that can influence the initiation and progression of carcinogenesis, and could act favourably on pathways implied in cell proliferation, apoptosis and metastasis (14-16).

Oral squamous cell carcinoma (OSCC) is one of the most prevalent cancer worldwide [incidence rate: 4 per 100.000 worldwide], and accounts for 90% of all malignancies of the oral cavity (17). Main risk factors of OSCC are tobacco and alcohol consumption, human papillomavirus infection and presence of oral potentially malignant disorders (18). Moreover, although treatments have improved over the years, prognosis of oral cancer is still poor (19).

It is noteworthy that in countries where populations of diverse ethnic groups co-exist, differences have been observed in terms of incidence of oral cancer (20). In this regard, when comparing between countries, the incidence rate of oral cancer in Mexico is 1.5, while in the USA it is much higher [4.3] (21), similar to what has been found when classifying by race and ethnicity in the later, where the rate of new cases of oral cancer is lower in Hispanics [4.62], including Mexicans, as compared to white people [9.53] (22). Diet could be considered as a possible factor to explain, at least partly, these differences. Eating chili peppers is a cultural tradition in Mexico and one of the most consumed foods included in their diet. In addition, Mexico holds one of the largest annual production (3.28 MT) and consumption (2.33 MT) of chili pepper (22); therefore, a high consumption of chili may be related to a low incidence of oral cancer.

The objective of this systematic review is to explore if there is evidence of a possible relationship between capsaicin intake and the incidence of oral squamous cell carcinoma, and discuss such association.

## Material and Methods

- Information sources and search strategy

The design of this study fulfils the PRISMA guidelines (23). We performed a systematic bibliographic research in PubMed [US National Gallery of Medicine], Web of Science and Scopus databases, whose strategy consisted of different combinations of the following keywords: capsaicin, chili, capsazepine, “oral cancer,” “oral carcinoma", "oral squamous cell carcinoma” and oscc (capsaicin AND "oral cancer”; capsaicin AND "oral carcinoma"; capsaicin AND "oral squamous cell carcinoma"; capsaicin AND oscc; chili AND "oral cancer”; chili AND "oral carcinoma"; chili AND "oral squamous cell carcinoma"; chili AND oscc; capsazepine AND "oral cancer”; capsazepine AND "oral carcinoma"; capsazepine AND "oral squamous cell carcinoma"; capsazepine AND oscc). All referenced articles were also screened for further manual inclusion. This review was not registered at any platform for systematic review.

- Inclusion and exclusion criteria

Using PICOs criteria (Table 1), in this review we address the relationship between capsaicin intake and oral carcinogenesis. PICO question was: oral cancer (population), treatment with capsaicin or analogues (intervention), oral cancer without exposure to capsaicin or analogues (comparison), to assess the effect of capsaicin in the development of oral squamous cell carcinoma (outcome) (23).

The inclusion criteria for the articles were: 1) studies published up to April 2020, 2) studies written in English or Spanish, and 3) experimental studies (in vitro and *in vivo*). Meanwhile, exclusion criteria were: 1) previous reviews, 2) studies that did not investigate the oral carcinogenesis, and 3) studies that did not use capsaicin as therapeutic agent.

**Table 1:**
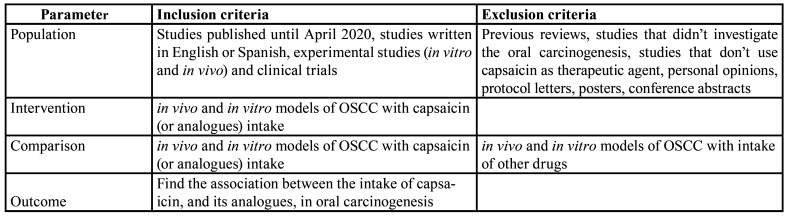
PICO criteria (participants/population, interventions, comparisons, outcomes, study design).

- Study selection and data extraction

The bibliographic research was performed by two independent reviewers (AMS and ILIM). All titles and abstracts that met the search criteria were red and then, the potentially eligible articles were analysed for their inclusion. Any disagreement between them was solved by a third and fourth reviewer (AMT or JMAU) to minimize bias of inclusion. Data from the included studies was collected by two reviewers (AMS and ILIM) and cross-checked by another (AMT or JMAU) to guarantee integrity of contents.

The information extracted from each study was: the author and year of publication, type of oral squamous cell carcinoma model (cell line and animal), number of cases, type of capsaicin intake, effect of capsaicin intake on oral carcinogenesis (incidence of epithelial dysplasia and oral cancer, epithelial-mesenchymal transition, cell proliferation, cell invasiveness, cell migration, apoptosis, chemoprevention, etc.).

- Risk of bias and quality assessment of the studies

OHAT Risk-of bias tool was used, for both *in vitro* and *in vivo* studies, to evaluate their methodological quality (24). OHAT risk of-bias rating is an effective approach that evaluates 11 different domains and 5 types of bias [selection, performance, attrition/ exclusion, detection and selective reporting]. The system for answering each question requires reviewers to choose between definitely low/ probably low/ probably high/ definitely high risk of bias.

In general, the methodological quality of the studies was good, and OHAT tool showed that risk of bias was probably low (24). Some questions of the selection and performance criteria were not reported by the authors; however, these items were not relevant and did not modify the overall risk of bias assessment.

## Results

- Bibliographical research

**Figure 1:**
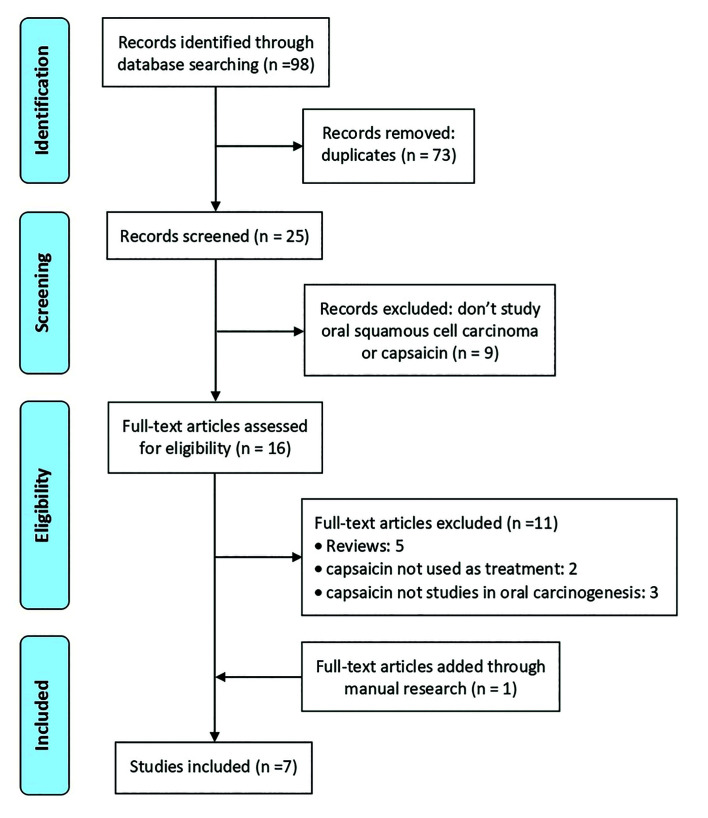
PRISMA flowchart. Synthesis of the bibliographical analysis.

We identified 98 records in the initial database search, out of which 73 were eliminated because were duplicates. After the first screening, another 4 records were excluded because they did not study oral squamous cell carcinoma and 5 more because they did not investigate about capsaicin. Thus, only 16 records were eligible for analysis; of these, 5 previous reviews were also removed, as well as 2 other studies that did not use capsaicin as therapeutic agent, and 3 that did not study the role of capsaicin in oral carcinogenesis. At the end, we added 1 article through manual research leaving the final number in 7 studies selected for the systematic review (6,25-30). Main data of the studies are shown in Table 2. The flowchart of the selection process is presented in Fig. 1.

**Table 2:**
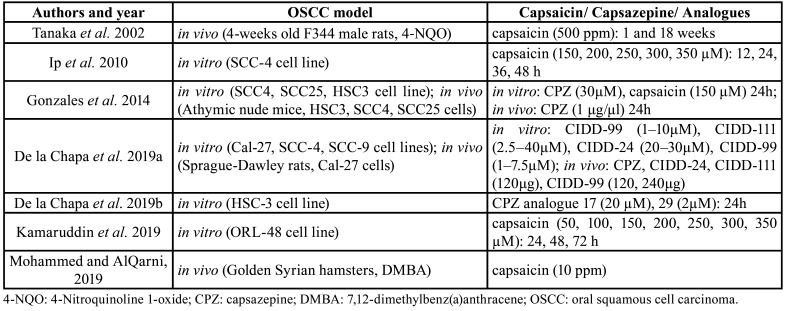
Main data of the included studies.

- Individual studies

Three of the 7 studies included in our analysis were *in vitro* (25,28-29), 2 *in vivo* (6,30) and 2 both *in vitro* and *in vivo* (26-27).

In vitro studies

Ip *et al*. (25) were the first to study whether different doses of capsaicin could induce apoptosis in tongue cancer cells. They observed that 300 µM capsaicin decreased the levels of mitochondrial membrane potential [calcium influx] and increased the reactive oxygen species [ROS]. An increase of AIF, cytochrome c, active-caspase-9, Bax, CHOP, Fas and active-caspase-8, and a decrease of pro-caspase-3 and Bid was also seen, all of which led to apoptosis. In addition, 350 µM capsaicin also decreased the percentage of viable cells, due to arrest of cell cycle at G0/G1 stage [dose-dependent]; and 400 µM capsaicin induced DNA condensation, damage and fragmentation.

De la Chapa *et al*. (28) developed potent analogues based upon capsazepine [CPZ] pharmacophore and structure-activity relationships [SAR] across analogues. Two of these, at different doses, had significant anti-proliferative effects in oral cancer cells.

Kamaruddin *et al*. (29) also investigated the apoptotic effect of capsaicin on human oral cancer cells. They saw that increasing concentrations of capsaicin were more cytotoxic to malignant cells. Capsaicin-induced apoptosis occurred through the shift in cell population from early apoptosis (24 h) to late apoptosis (48 and 72 h). It also increased the activity of caspase-3-7-9 and mitochondrial membrane potential. Therefore, a lower percentage of viable malignant cells was seen in capsaicin treated cells after G1-phase, in comparison to control cells. These results indicated that cell cycle arrest of capsaicin-treated cells is associated with the antiproliferative effect in a time-dependent manner.

In vivo studies

The effects of dietary administration of oral capsaicin on an animal tongue cancer model was firstly investigated by Tanaka *et al*. (6). These authors demonstrated a lower incidence of oral epithelial dysplasia (OED) and carcinoma in capsaicin-treated rats. Apoptotic index (ssDNA) was higher in the capsaicin group, while proliferative index (PCNA) was lower.

Mohammed and AlQarni (30) also investigated the anti-proliferative effect of capsaicin in OED and carcinogenesis. Capsaicin treated animals had a lower incidence and severity of OED (lower immunoexpression of Bcl-2).

In vitro and *in vivo* studies

Gonzales *et al*. (26) tested diverse cell lines with different doses of CPZ and capsaicin. An 80% reduction in cell viability was observed with CPZ and capsaicin together. Capsazepine also induced apoptotic activity (accumulation of subG1 cells, increase of ROS and c-PARP) to all OSCC cell lines, even at low doses (30 μM). However, this effect changed among cell lines, with more HSC3 and SCC25 cells in apoptosis (30 μM) or cell death (60 μM), than SCC4 cells at the same concentrations. These authors (26) also carried out an *in vivo* experiment with mice, and they found that CPZ treated HSC3 tumours did not enlarge as much as control group, and dramatically shrank in size, similar to SCC25 tumours, which displayed a marked reduction in tumour volume following treatment with CPZ (50.5%). In contrast, SCC4 control xenograft tumours grew nearly two-fold greater than CPZ treated cases, and showed more apoptotic Figures.

De la Chapa *et al*. (27) demonstrated the apoptotic effect of both CPZ and CPZ analogs on various malignant cells by means of S-phase block, mitochondrial dysfunction and ROS-mediated c-PARP increase. CIDD-99 (500 μM) also caused an anti-proliferative effect against Cal-27 cells when used with cisplatin, gefitinib, and radiation. The efficacy of these analogues was also assessed *in vivo*, all of which reduced tumour volume. Furthermore, in analog CIDD-99 treated rats, there were even no tumour cells remaining in some cases.

## Discussion

Oral carcinogenesis involves a series of steps which are controlled by many factors, including genetic, metabolic and environmental ones. About 30-35% of carcinomas are initiated due to dietary factors (31); however, many types of compounds have also been studied as chemopreventive agents.

The link between capsaicin and cancer has long been studied (32). Initial investigations suggested that continuous intake of capsaicin increased the risk to develop some carcinomas of the digestive tract, including gastric cancer. However, it was later discovered that capsaicin might act, in fact, as a chemopreventive agent, altering the microsomal function of several enzymes, which are key for the metabolic activation and detoxification of multiple mutagens.

The first authors who reported the anticarcinogenic properties of capsaicin were Modly *et al*. (33), who indicated that this substance inhibited the metabolism of many polycyclic aromatic hydrocarbons like benzo[a]pyrene 3 and suppressed their binding to human DNA. Many studies have proven that this effect is obtained through the modulation of cytochrome P450 and NADPH dependent activities (34).

New discoveries have been made in the last years regarding the antiproliferative and apoptotic effect of capsaicin on human colon and oesophageal carcinoma (35). It has been demonstrated that capsaicin supresses both intrinsic and extrinsic tumour signalling pathways, which are necessary for the invasion and migration of malignant cells (36).

It is of great interest to know the specific effect of this compound on carcinomas of the upper aerodigestive tract. Previous studies (37-38) have proven that capsaicin supresses the growth of gastric cancer through downregulation of several pathways (NADPH, ERK 1/2, p38 MAPK, JNK), inhibition of inflammatory molecules (IL-6) and increase of apoptotic molecules (caspase-3, p53). On the other hand, capsaicin also induces G1 arrest and apoptosis of nasopharyngeal cancer cells through mitochondrial depolarization and acting on specific pathways (PI3K/mTOR) and molecules (caspase-3, Bcl-2) (39). In addition, it retrains the invasion and migration of malignant oesophagus cells by inhibition of MAPK signalling pathway, intracellular stress and promotion of p53 (40).

The reason behind this review was to acknowledge the link between capsaicin and oral cancer (8). Previous studies showed molecular evidences of oral cell proliferation inhibition associated to other compounds, such as lycopene, green tea polyphenols, resveratrol and curcumin (41). Apoptosis of oral malignant cells has also been related to lycopene, quercetin, epigallocatequin gallate, theaflavin-3 gallate, garlic and ginger (42-43).

**Figure 2:**
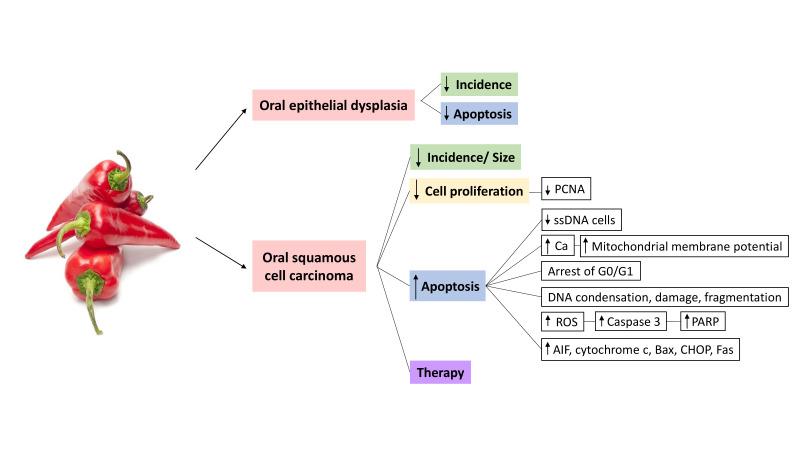
Oral carcinogenesis processes in which capsaicin/capsazepine/analogues may be implicated.

According to our search, all the studies included in this review (6,25-30) confirm that capsaicin must be considered as a chemopreventive agent for oral cancer (Fig. 2) check Supplement1 (http://www.medicinaoral.com/medoralfree01/aop/24570_supplement1.pdf). Since the original report, it was suggested that continuous intake of capsaicin decreased the incidence of oral cancer, because it stopped the proliferative activity of malignant cells and increased their apoptosis (6). Moreover, different studies have observed a reduction on tumour size in mice treated with CPZ (26), and even absence of malignant cells in rats treated with CPZ (27).

This cytotoxic effect of capsaicin has been demonstrated and reinforced with different techniques throughout the years, and it seems to be time and dose dependent; however, since low doses of capsaicin continue to be chemopreventive, multiple *in vitro* studies have been carried out in order to uncover its molecular mechanisms. Ip *et al*. (25) pointed out that capsaicin induces G0/G1 cell cycle arrest and apoptosis in SCC-4 cells, through an increase of ROS, Ca2+ and caspase-9. Also, it produces the overexpression of several molecules of cellular stress that lead to apoptosis (AIF, cytochrome c, Bax, CHOP, Fas). The same apoptotic pathway was observed by Kamaruddin *et al*. (29), with less viable cells after G1-phase in the capsaicin treated group, due to an increase of caspases and mitochondrial depolarization, and also similar results were found by de la Chapa *et al*. (27), who reported an increase of ROS-mediated c-PARP.

It is noteworthy that, in spite of using different cell lines, capsaicin doses and duration of treatments, both capsaicin and CPZ trigger the same alterations on the malignant cells. We consider that these results indicate that the antiproliferative effect of capsaicin could be linked to apoptosis. Interestingly, some studies showed that CPZ has a stronger cytotoxic activity than capsaicin, since more cells underwent apoptosis and showed phenotypic changes (26). Furthermore, CPZ without capsaicin also decreases cell proliferation and tumour size, and even promotes the effect of chemo and radiotherapy (27-28).

Another aspect of great interest is the link between capsaicin and OED (6,30). To date, histopathological analysis is the gold standard technique to make a diagnosis of oral cancer, while presence of OED represents the key prognostic factor to predict malignant transformation of a lesion in the oral mucosa; thus, its recognition and graduation is very important for primary and secondary prevention of oral cancer. In this sense, Tanaka *et al*.(6) found that capsaicin-treated animals had a lower incidence of OED, which later Mohammed and AlQarni (30) proved to be related to a lower immunoexpresion of anti-apoptotic biomarker Bcl-2.

**Figure 3:**
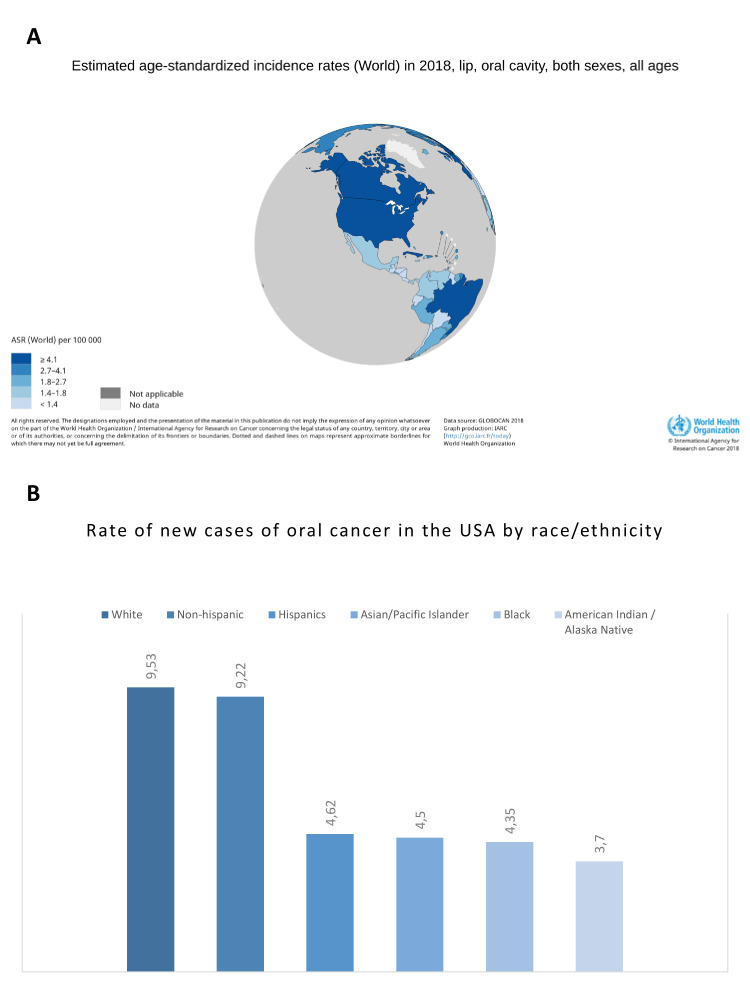
Incidence rates of oral cancer. A) Worldwide, see the differences between Mexico (1.5) and USA (4.3) (WHO, 2018); B) By race and ethnicity in the USA population (NIH, 2017).

Considering all the available information, we believe dietary consumption of capsaicin could provide a chemopreventive effect for oral squamous cell carcinoma. We think this protective property may explain why the incidence of oral cancer upon chili consumers is lower. In fact, peppers cover 1.93 million ha of crop-growing surface areas worldwide, and Mexico is the second largest pepper producer in the world (2.3 million) (1). Thus, intake of capsaicin is an important sociocultural element in some populations. Although indirectly, there is epidemiological evidence suggesting that the protective effect of capsaicin could be responsible, at least partially, for the disparities observed in the incidence of oral cancer among different ethnic groups living in the same population, such as the USA, where the rate of new cases of oral cancer is significantly lower in Hispanics than in white people (21) (Fig. 3).

Nevertheless, more studies are needed to further understand the mechanism by which these compounds prevent oral carcinogenesis, and to develop novel therapeutic agents for clinical application against oral cancer.

## Conclusions

In summary, this study gives a comprehensive view of the relationship between capsaicin and oral carcinogenesis. Both capsaicin and capsazepine are chili substances that may prevent the development of oral epithelial dysplasia and oral squamous cell carcinoma. The steps in which these compounds could have a chemopreventive activity against oral cancer are: inhibition of malignant cell proliferation and increase of malignant cell apoptosis. These findings elucidate why dietary intake of capsaicin could explain the existing differences in the prevalence of OSCC in certain world populations. Future research should focus on humans, to investigate the relationship between consumption of specific foods such as chili (capsaicin) and the prevalence of oral cancer, through well-designed studies (patient-clinician communication, food frequency questionnaires, etc.).
